# Compartment syndrome following intramuscular self-injection of kerosene and rodenticide: A case report

**DOI:** 10.1016/j.ijscr.2021.106233

**Published:** 2021-07-22

**Authors:** Daniel Asiimwe, Walufu Ivan Egesa, William Mugowa Waibi, Dickson Kajoba, Patrick Kumbowi Kumbakulu

**Affiliations:** a Department of Surgery, Faculty of Clinical Medicine and Dentistry, Kampala International University, Uganda; b Department of Paediatrics and Child Health, Faculty of Clinical Medicine and Dentistry, Kampala International University, Uganda

**Keywords:** Kerosene, Rodenticide, Suicide, Compartment syndrome, Case report

## Abstract

**Introduction and importance:**

Kerosene and rodenticides are used in many households in developing countries. This case report aims to discuss the progression and management of a patient with intentional kerosene and rodenticide poisoning. To our knowledge, this is the first documented case of blended kerosene-rodenticide poisoning in medical literature.

**Case presentation:**

This report describes a 23-year-old man who survived after intramuscular self-injection of 5 ml of kerosene mixed with a rodenticide into his left upper limb, with intent to commit suicide. He was admitted to our hospital following a convulsion and brief loss of consciousness. Compartment syndrome developed within 24 h of admission, necessitating urgent fasciotomy, repeated surgical debridement, limb elevation, wound cleaning and dressing, in addition to intravenous fluids, antibiotics, and close observation. Blood transfusion, phytomenadione (vitamin K1), tetanus toxoid, and analgesics were recommended. The patient also received physiotherapy, and was treated for depression. The limb healed completely, with contractures at the left wrist joint.

**Clinical discussion:**

Injected kerosene and rodenticide may result in compartment syndrome and variable local and systemic complications which require multifaceted care and a prolonged follow-up period.

**Conclusion:**

Seemingly minor injuries at presentation may quickly progress into considerable complications such as compartment syndrome. It is imperative that physicians comprehensively investigate patients with poisoning for multiorgan dysfunction. Anticipation of local and systemic complications of injected poisons and timely medical and surgical intervention is life-saving.

## Highlights

• Injection with kerosene mixed with rodenticide has not been documented in medical literature.
• Clinicians should anticipate local and systemic complications of injected poison.
• Treat compartment syndrome with fasciotomy, repeated debridement, and supportive measures.
• Early surgical intervention is life-saving, and leads to limb salvage.

## 1 Introduction

Kerosene is a liquid hydrocarbon that is mainly used as a solvent fuel for cooking and lighting in many households in developing countries. Experience has been gained with ingested and inhaled hydrocarbons, being the common routes of accidental or intentional poisoning. Intentional poisoning is mostly observed among adolescents and adults [1], whereas accidental injection may occur as part of recreational abuse or in an industrial setting [2]. Injection with kerosene is an uncommon occurrence, often linked to suicidal attempts, and mostly involving the upper extremities [1], [3]. Males are predominantly more affected compared to females [1].

In the same manner, rodenticides (rat poisons) are widely used to control rodent populations, increasing the risk of intoxication and suicide. Besides oral intake, rodenticides are easily absorbed through the skin, because they are lipophilic [4]. According to the American Association of Poison Control Centers, majority of human rodenticide exposures are due to long-acting anticoagulants, bromethalin, phosphides, cholecalciferol, and warfarin [5]. In this report, we describe a case of intentional administration of kerosene mixed with an unidentified rodenticide, and also review related literature. This study was written in compliance with the SCARE 2020 criteria [6].

## 2 Case presentation

A 23-year-old male presented to a referral hospital following attempted suicide by intramuscular self-injection of approximately 5 ml of kerosene blended with rodenticide into two sites in his left upper limb. This act was his first, and was preceded by a relationship conflict. There was no history of poison ingestion, previously diagnosed mental health disorder, underlying chronic medical illness, or regular-use medication, and no significant previous surgical history. He subsequently developed a seizure, lost consciousness, and was brought by his neighbours to the accident and emergency unit within 2 h of poisoning.

**Fig. 1:**
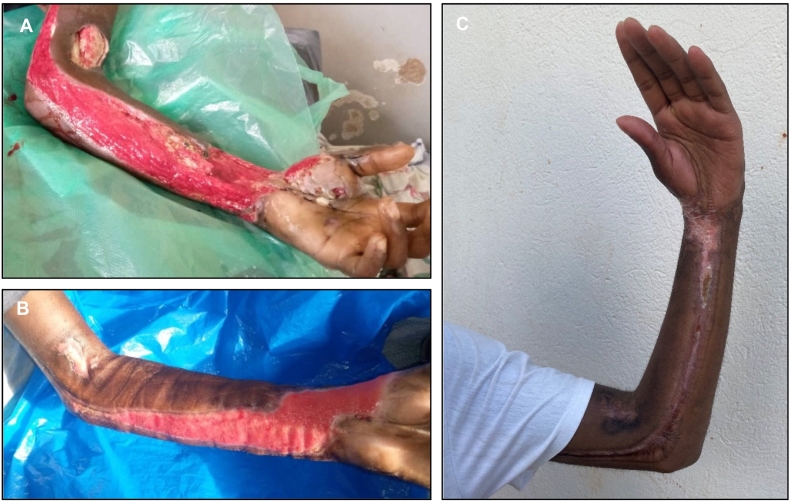
Photographs of the left upper extremity on day-19 (A) and day-63 (B) after fasciotomy and three sessions of extensive surgical debridement. Photograph of the same limb demonstrating healing, with wrist contractures on day-155 after fasciotomy (C).

At admission to the medical ward, he was conscious, alert, and had no focal neurological signs. He experienced abdominal pain and generalized body aches. Local examination revealed needle-entry marks over the left wrist and cubital fossa. Examination of other extremities was normal. Respiratory and cardiovascular system assessment was normal; he was afebrile; but abdominal assessment revealed generalized tenderness. His blood pressure was 113/86 mmHg, pulse rate 115 bpm, oxygen saturation 96% in room air, with a random blood glucose level of 8.0 mmol/L. At admission, the attending physician recommended nil per os for at least 24 h, intravenous normal saline and dextrose 3 l were administered in 24 h, and analgesics were given. A complete blood count revealed a white blood cell count of 3880 cell/μl, haemoglobin level of 17.4 g/dl, and platelet count of 177,000/μl. The following day, he had developed marked swelling of the left forearm and hand; the skin had blisters and was shiny; and cold fingers with limited movement were noted. On the basis of these signs, a diagnosis of compartment syndrome of the left upper extremity was reached, which warranted urgent fasciotomy and surgical debridement. This prompted his transfer to the surgical department, followed by fasciotomy extension and repeat surgical debridement on day-8 and day-17, with removal of devitalized skin and subcutaneous tissue. Fig. 1A shows a photograph of the left upper limb on day-19 post-fasciotomy. Surgical procedures were accomplished using intramuscular pethidine, and were not associated with prolonged bleeding. Limb elevation was performed, and wound dressing was done using vinegar and vaseline gauze. Following the third session of debridement, the patient developed hemorrhage originating from the anterior proximal forearm. Pressure dressing was applied, intramuscular phytomenadione (vitamin K1) was administered, and haemostasis was achieved. Blood grouping and crossmatching was done (O rhesus D positive), and he was subsequently transfused with 2 units of whole blood. The pretransfusion haemoglobin level was 4.4 g/dl. No other laboratory or radiological investigations were requested due to financial challenges, and maintenance therapy with vitamin K1 was not recommended.

During the course of hospitalization, no other history of unexplained bleeding was documented. Intravenous antibiotics were administered (Ampiclox 1 g 8 hourly, and Metronidazole 500 mg 8 hourly, Meropenem 1 g 8 hourly), intravenous crystalloids, analgesics (intravenous tramadol 100 mg 8 hourly, diclofenac 75 mg 8 hourly, oral morphine 10 ml 8 hourly, paracetamol tabs 1 g 8 hourly), bisacodyl tablets 10 mg nocte, haematinics, and oral fluid intake. He also received evaluation by a physiotherapist, and mental health assessment by a psychiatrist. A diagnosis of depression was made, and oral amitriptyline was prescribed.

Gradually, granulation tissue formed, and he was discharged 42 days after admission. Home-based follow-up on day-63 revealed granulation tissue formation with slopy edges of the wound (Fig. 1B). Wound care continued, and on day-155 after fasciotomy, the wounds had healed, but with contractures at the left wrist (Fig. 1C). The patient had no other concerns, and was able to perform routine activities, including riding a motorcycle. Continuation of physiotherapy was recommended.

## 3 Discussion

While ingestion of hydrocarbons and rodenticides is common, parenteral administration of a mixture of a hydrocarbon and rodenticide has not been documented before in medical literature. Attempted suicide by tissue injection of hydrocarbons is often linked to mental disorders, including depression, schizophrenia, and drug addiction [3], [7]. The most common site is the anterior forearm, through the subcutaneous, intramuscular, intravenous, or mixed routes [3], [8]. In this case, the intramuscular route was used.

The clinical presentation of hydrocarbon poisoning depends on the route of exposure. However, clinical manifestations do not significantly differ for parenteral administration of hydrocarbon [3]. Ingestion and inhalation often result in symptoms of chemical pneumonitis, whereas injection with hydrocarbons can result in significant local and systemic morbidities such as cellulitis, thrombophlebitis, compartment syndrome, necrotizing fasciitis, abscess formation, arrhythmia, and reversible pulmonary oedema [1], [3], [7], [8], [9]. The neurovascular status of affected extremities should therefore be monitored for early signs of compartment syndrome [3]. Injection of kerosene or its derivatives may also cause agitation, drowsiness, lethargy, and fever [1], [8], [10]. Kósa [11] described a patient who developed tonic-clonic convulsions, loss of consciousness, and died shortly after accidental intravenous injection of petrol by a medical doctor. Similarly, Amiri and colleagues [1] described 10 intravenous drug addicts, 8 of whom lost consciousness after intravenous administration of kerosene. Our patient developed a convulsion and lost consciousness, but never manifested with respiratory or other systemic symptoms. He was diagnosed with compartment syndrome which developed within 24 h of hospitalization.

There is no specific antidote for hydrocarbon toxicity. Therefore, symptom-based and goal- directed supportive care remains the mainstay of therapy. Elevation and immobilization of the affected extremity, intravenous antibiotics, careful monitoring for local and systemic complications are key aspects in management. Some authors [8] have reported the use of intravenous steroids, although this has not been extensively studied. Researchers have suggested surgical intervention by fasciotomy and repeated debridement as the definitive treatment for patients who develop compartment syndrome and necrotizing fasciitis, with or without need for skin grafting. In addition, abscesses should be drained [3]. As with many other reported cases, this aggressive approach averts the need for limb amputation [3], [12]. Survival after intravenous administration of up to 30 ml of kerosene or its derivatives has been documented in several case reports [3], [8], [10], but administration of more than 5 ml of kerosene is associated with significantly higher likelihood of mortality [1].

Long-term follow up is required, because these patients may develop contractures, neurological deficits following nerve entrapment or ischemia, and oedema following vascular insufficiency [3]. As in the present case, contractures developed, requiring reconstructive surgery and physiotherapy. Mental health evaluation and subsequent follow-us should be emphasized, since repeat suicidal attempt may occur [3], [12].

We were unable to determine the specific type and quantity of rodenticide that the patient added to kerosene. When used to commit suicide, rodenticides such as cholecalciferol, metal phosphides, anticoagulants, bromethalin, among others, are frequently administered orally [4], [13], [14], [15], and thus, literature on parenterally administered rodenticides is scanty. The wide array of clinical manifestations that are unique to different types of rodenticides makes it possible for physicians to narrow down the potential rodenticide. First generation (warfarin) and second-generation anticoagulant rodenticides (brodifacoum, bromadiolone, difenacoum, flocoumafen and difethialone) can cause epistaxis, haematuria, haematochezia, massive pulmonary hemorrhage, intracranial hemorrhage, easy bruising, and anaemia, due to inhibition of the enzyme vitamin K epoxide reductase, which results in inactivation of vitamin K dependent clotting factors II, VII, IX, and X [4], [15], [16]. Prolonged patient monitoring is warranted, given that the plasma half-life of second-generation anticoagulant rodenticides (AR) lies between 16 and 220 days, and that coagulopathy may be observed several days after intoxication [4], [16]. However, our patient did not develop coagulopathy during the 5-month period of follow up, perhaps because he may have used a less potent rodenticide, or lower dosage of rodenticide. Nonetheless, some rodenticides such as arsenic and fluoroacetamide may lead to neurological toxicity, manifesting with seizures and coma, while others such as zinc phosphide may cause renal failure and cardiorespiratory problems (shock, congestive heart failure), and pulmonary oedema, which were absent in this patient [14]. The multiorgan toxic effects of metal phosphides are a result of toxic phosphine gas that is liberated after hydrolysis by gastric acid [14], a process which requires oral ingestion. None life-threatening injection of up to 5 ml of zinc phosphide has been documented [17].

Considering that the anticoagulant effects of AR can be reversed by administration of vitamin K1 and fresh frozen plasma [16], our patient received vitamin K1 and whole blood transfusion only after he started bleeding. Meanwhile, we did not attribute his bleeding episode to AR exposure, because it occurred following debridement, and we did not determine the prothrombin time (PT) and international normalized ratio (INR). We therefore recommend that physicians attending to patients with rodenticide poisoning should evaluate and monitor for coagulopathy and organ dysfunction.

## 4 Conclusion

Deliberate self-injection with a hydrocarbon-rodenticide mixture is a previously undocumented phenomenon, which calls for evaluation for an underlying mental health problem. Physicians should be enlightened about the potential devastating complications of hydrocarbon and rodenticide intoxication, and need to contact an expert toxicologist. In consideration of the diversity of rodenticides, physicians should make great effort to identify the specific poison, in order to guide treatment decisions. Limb salvage and recovery was possible following urgent fasciotomy, repeated surgical debridement, and meticulous supportive care.

## Sources of funding

None.

## Ethical approval

Not required. Single case reports are exempt from ethical approval in our institution.

## Consent for publication

Written informed consent to publish this case report and accompanying images was obtained from the patient. A copy of the written consent is available for review by the Editor-in-Chief of this journal on request.

## Authors' contributions

DA participated in patient care. DA, WIE, WMW, DK, and PKK collected clinical information and followed-up the patient. WIE drafted the original manuscript. DA and WMW reviewed and edited the manuscript. All authors read and approved the final manuscript.

## Research registration number

Not applicable.

## Guarantor

Daniel Asiimwe.

Walufu Ivan Egesa.

## Provenance and peer review

Not commissioned, externally peer-reviewed.

## Declaration of competing interest

There are no conflicts of interest.
